# Critical Role of Rubber Functionalities on the Mechanical and Electrical Responses of Carbon Nanotube-Based Electroactive Rubber Composites

**DOI:** 10.3390/polym17020127

**Published:** 2025-01-07

**Authors:** Md Najib Alam, Siraj Azam, Jongwan Yun, Sang-Shin Park

**Affiliations:** School of Mechanical Engineering, Yeungnam University, 280, Daehak-ro, Gyeongsan 38541, Republic of Korea; mdnajib.alam3@gmail.com (M.N.A.); sirajazam@gmail.com (S.A.); unki211@naver.com (J.Y.)

**Keywords:** functional rubber, dielectric elastomers, mechanical properties, thermodynamics, filler–polymer interactions, electromechanical sensors and actuators

## Abstract

Carbon nanomaterials, particularly carbon nanotubes (CNTs), are widely used as reinforcing fillers in rubber composites for advanced mechanical and electrical applications. However, the influence of rubber functionality and its interactions with CNTs remains underexplored. This study investigates electroactive elastomeric composites fabricated with CNTs in two common diene rubbers: natural rubber (NR) and acrylonitrile-butadiene rubber (NBR), each with distinct functionalities. For NR-based composites containing 2 vol% CNTs, mechanical properties, such as elastic modulus (2.24 MPa), tensile strength (12.48 MPa), and fracture toughness (26.92 MJ/m^3^), show significant improvements of 125%, 215%, and 164%, respectively, compared to unfilled rubber. Similarly, for NBR-based composites, the elastic modulus (5.46 MPa), tensile strength (13.47 MPa), and fracture toughness (82.89 MJ/m^3^) increase by 94%, 22%, and 65%, respectively, over the unfilled system. Although NBR-based composites exhibit higher mechanical properties, NR systems show more significant improvements, suggesting stronger chemical bonding between NR chains and CNTs, as evidenced by dynamic mechanical, X-ray diffraction, thermogravimetric, and thermodynamic analyses. The NBR-based composite at 1 vol% CNT content exhibits 261% higher piezoresistive strain sensitivity (GF = 65 at 0% ≤ Δε ≤ 200%) compared to the NR-based composite (GF = 18 at 0% ≤ Δε ≤ 200%). The highest gauge factor of 39,125 (1000% ≤ Δε ≤ 1220) was achieved in NBR-based composites with 1 vol% CNT content. However, 1.5 vol% CNT content in NBR provides better strain sensitivity and linearity than other composites. Additionally, NBR demonstrates superior electromechanical actuation properties, with 1317% higher actuation displacement and 276% higher electromechanical pressure compared to NR at an applied electric field of 12 kV. Due to the stronger chemical bonding between the rubber and CNT, NR-based composites are more suitable for dynamic mechanical applications. In contrast, NBR-based CNT composites are ideal for stretchable electromechanical sensors and actuators, owing to the high dielectric constant and polarizable functional groups in NBR.

## 1. Introduction

Dielectric elastomer-based nanocomposites have recently garnered significant attention in the fields of science, engineering, and technology [1,2]. Modern engineering devices require high stretchability, durability, conductivity, and robustness. Rubber polymers exhibit these features due to their low glass transition temperatures and excellent dynamic properties. However, despite these advantages, rubbers often present drawbacks, such as poor thermal, electrical, and magnetic properties, which limit their application as electroactive or magnetoactive materials [3,4]. To enhance these properties, rubber can be blended with particulate fillers that provide special functionalities [5,6]. For instance, blending rubber with magnetic fillers can introduce magnetic properties into the composite [7,8]. Similarly, incorporating thermal or electrical conducting fillers can significantly improve its thermal and electrical properties [9]. Additionally, the fabrication of rubber composites is cost-effective, straightforward, and reliable, making it a viable option for industrial applications.

Different types of reinforcing and non-reinforcing fillers can be used to enhance the desired properties of rubber composites. Filler morphology, surface characteristics, and filler dispersion are critical factors that govern the ultimate properties of these composites [10]. Inorganic fillers, such as silica, clay, and minerals, are well-known as effective reinforcing fillers for rubbers [11]. However, to achieve improved properties, these fillers often require surface treatments [12], which can be expensive and time-consuming. For over a century, carbon materials, such as carbon blacks, have been utilized as reinforcing fillers in tires [13]. Carbon materials exhibit strong π–π interactions, in addition to van der Waals forces with diene rubbers, making them suitable for reinforcement without requiring surface treatments. Furthermore, carbon materials significantly enhance heat buildup, abrasion, and aging resistance properties [14,15]. Consequently, carbon materials continue to be regarded as effective reinforcing fillers for commercial diene rubbers. However, due to their low aspect ratio and surface area, carbon blacks require larger quantities to achieve effective reinforcement. Among various carbon materials, graphitic nanocarbons, such as nanographite, graphene, and carbon nanotubes (CNTs), significantly improve the thermal and electrical properties of rubbers, making them ideal for advanced mechanical and electrical applications [16]. For example, multiwalled carbon nanotubes (CNTs) exhibit electrical conductivities of approximately 20–2 × 10^7^ S/m [17] and thermal conductivity values around 3000 W/mK [18], which are suitable for preparing rubber composites with exceptional thermal and electrical conductivities. Recently, CNT-based elastomer nanocomposites have found numerous applications in flexible devices for strain sensing [19], robotics [20], energy harvesting [21], electromagnetic interference shielding [22], and other fields [23]. The properties of these composites depend on various factors, including filler morphology, dispersion, and filler–polymer interactions. For many advanced applications, mechanical stability is crucial while maintaining uniform conductivity in the nanocomposites [24,25,26]. Elastomers, such as natural rubber [27], styrene-butadiene-styrene rubber [28], carboxylated acrylonitrile-butadiene rubber [29], and silicone rubber [30,31,32,33], have been used to fabricate CNT-based nanocomposites, and their properties have been extensively investigated. Most studies have focused on properties related to filler characteristics, morphology, and distribution within the rubber matrix. Some researchers have developed strain-sensing composites based on natural rubber and acrylonitrile-butadiene rubber, incorporating conducting carbon materials [34,35,36]. From the reported gauge factor values, it has been observed that nitrile rubber-based composites exhibit better sensitivities compared to natural rubber-based composites [34,35,36]. Since strain sensitivities were studied separately, there has been limited exploration of how sensitivities vary with different functional rubbers. Several types of strain-sensing composites can be fabricated with conducting fillers embedded in dielectric polymer materials [37]. Among these, resistive and capacitive types are the most widely studied [37]. It has been demonstrated that embedding conducting materials in insulating materials leads to enhanced dielectric properties due to the formation of micro capacitors [38,39,40,41]. Since different rubbers have varying dielectric constant values, the capacitance of rubber composites can differ, which in turn, affects their strain sensitivities. In addition to electrical applications, acrylonitrile-butadiene rubber is widely used in membrane and oil field technologies due to its excellent oil resistance properties [42,43].

Although electroactive rubber composites have been developed and continue to be improved for enhanced sensitivity and mechanical robustness, it is well known that different rubbers exhibit varying affinities for filler materials, leading to differences in filler dispersion. Filler dispersion and, ultimately, filler–polymer interactions can result in variations in mechanical properties. Moreover, while rubber-based composites have been shown to be electroactive, the effects of functional groups on their performance have yet to be fully explored. Due to the dielectric variation and polarizability of the functional groups in rubber, these materials may exhibit different behaviors in the presence of an electric field. Some rubbers, such as acrylonitrile-butadiene rubber, contain polarizable nitrile functional groups that can rearrange their dipoles in the direction of the induced electric field, resulting in rubber chain contraction. These characteristics are particularly important for achieving variable stiffness in artificial muscles or soft robotics [44,45].

In this research, we aim to demonstrate that functional rubbers with different functional groups play a critical role in the properties of electroactive materials. To achieve this, we selected two different diene rubbers: natural rubber (cis-polyisoprene) and acrylonitrile-butadiene rubber (a copolymer of butadiene and acrylonitrile), each containing methyl and nitrile side chain functional groups, respectively. Due to differences in electronegativity and bonding environments, these rubbers exhibit distinct dipole moments. To create stretchable electroactive rubber composites, varying amounts of multiwalled carbon nanotubes (CNTs) were incorporated through solution blending. Diene rubbers are known for their high stretchability and outstanding fracture toughness, which make them ideal for producing stretchable thin films for various electronic applications. The physical, mechanical, and electroactive properties of the composites were thoroughly investigated and compared, with a focus on how the rubber functionalities influence these properties. The mechanism of filler–polymer interactions and their relationship with the observed properties is critically discussed. Finally, the composites were evaluated for electromechanical activities, such as strain sensing and actuation performance.

## 2. Materials and Methods

### 2.1. Materials

Two different types of rubber were used: natural rubber (NR, STR 5L, >95% cis-polyisoprene content) and acrylonitrile-butadiene rubber (NBR, KNB 35H, ~34% acrylonitrile content), both purchased from Kumho Petrochemicals, Seoul, Republic of Korea. Various rubber-grade curing additives, including zinc oxide, stearic acid, sulfur, accelerator tetramethyl thiuram disulfide (TMTD), and accelerator N-tert-butyl-benzothiazole sulfonamide (TBBS), were utilized to prepare the masterbatch rubber. The primary role of curing additives is to form crosslinks between the rubber chains, resulting in significant improvements in the elasticity and mechanical stability of the rubber compound. Masterbatches from different rubbers were prepared by mixing on a two-roll mill for 30 min using similar amounts of curing additives, expressed as parts per hundred rubber (phr). The compositions in the masterbatches were as follows: 100 g of rubber, 5 phr zinc oxide, 2 phr stearic acid, 1 phr TMTD, 1.75 phr TBBS, and 1.5 phr sulfur. The masterbatches were prepared using the same sequences and methods as described in previous studies [46,47]. Toluene (GR grade) was purchased from Daejung Chemicals & Metals, Siheung-si, Republic of Korea. The multiwalled carbon nanotube filler (CNT, CM-100, density = 1.74 g/cm^3^) was obtained from Hanwha Nanotech Corporation Ltd., Seoul, Republic of Korea.

**Figure 1:**
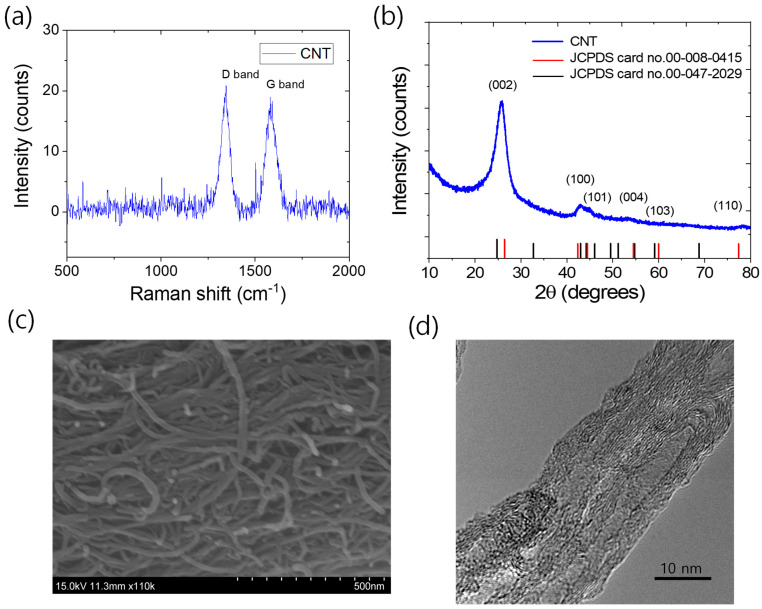
Characteristics of used carbon nanotubes. (**a**) Raman spectra, (**b**) XRD, (**c**) SEM image, and (**d**) TEM image.

Raman spectra, X-ray diffraction patterns, scanning electron microscope (SEM), and transmission electron microscope (TEM) images are shown in Figure 1a–d. The characteristic D band and G band peaks at ~1345 cm⁻^1^ and 1575 cm⁻^1^, respectively, and their relative intensity ratio in the Raman spectroscopy (Figure 1a) indicate that the CNT contains sp^2^ and sp^3^ carbon defects, which may exhibit chemical reactivity [48]. The X-ray diffraction pattern of the CNT corresponds well to the hexagonal graphite structure (JCPDS card no. 00-008-0415) with various planes, as shown in Figure 1b. However, some major peaks also match with the orthorhombic trans-polyacetylene crystal system (JCPDS card no. 00-047-2029), suggesting that the CNT may contain other carbonaceous impurities. The SEM image (Figure 1c) shows that the diameter of the CNT is less than 25 nm, and it exhibits a very high aspect ratio. The TEM image (Figure 1d) clearly shows that the CNT is multiwalled in structure.

### 2.2. Fabrication of CNT-Based Rubber Nanocomposites

To maintain uniform electrical conductivity in rubber nanocomposites, the solvent blending technique was employed instead of dry mixing [49]. In solvent mixing, the rubber chains are sufficiently free from intermolecular interactions, allowing the deposition of rubber molecules onto the filler surfaces. This process enhances the wettability of the filler surfaces by the rubber chains, thereby improving filler dispersion as well [34,35]. However, the environmental pollution associated with the use of toluene as a solvent can be mitigated through the proper handling of the solvent [46,47].

The method for fabricating CNT-based rubber composites is described elsewhere [33]. Initially, 25 g of masterbatch rubber was placed in a jar, and 100 mL of toluene was added and allowed to soak for approximately 24 h. After soaking, the rubber was homogenized by mechanical stirring to create a slurry. In a separate container, the required amount of CNT (0 to 2.5 vol%) was combined with 100 mL of toluene and placed in an ultrasonication bath for 30 min. The entire CNT solution was then added to the rubber slurry and mixed thoroughly with a mechanical stirrer for 10 min. The CNT-mixed rubber slurry was transferred to a flat tray and dried at 80 °C for approximately 24 h until it reached a constant weight. The dried compounded rubber was then placed in a mold and cured under compression at 150 °C for 15 min to ensure complete curing [49]. The cured samples were stored in a refrigerator to minimize the effects of environmental aging. Prior to the next characterization, the samples were brought to room temperature and allowed to rest for approximately 24 h. The different CNT concentrations in the masterbatch rubber are provided in Table 1.

### 2.3. Compressive and Tensile Mechanical Analyses

For compressive mechanical studies, cylindrical samples (height = 10 mm, diameter = 20 mm) were prepared. For tensile mechanical studies, dumbbell-shaped samples (ISO 37, type 2, gauge length = 25 mm) were punched from 1 mm thick vulcanized sheets. Static mechanical testing was conducted using a universal testing machine (UTM) equipped with a 1 kN load cell. The motor speeds of the UTM were set at 2 mm/min for compressive testing and 300 mm/min for tensile testing. For each specific value, the average of four test specimens is reported.

### 2.4. Dynamic Mechanical Studies

Dynamic mechanical thermal analyses were conducted using a dynamic mechanical analyzer (DMA Q800, New Castle, DE, USA) in tension mode, with a temperature range of −100 to +100 °C and a heating rate of 2 °C/min, in a nitrogen atmosphere. Rectangular test specimens with uniform dimensions (10 mm × 4 mm × 1 mm) were punched from the rubber sheets and used for the DMA analyses.

### 2.5. X-Ray Diffraction Studies of Rubber Composites

XRD analyses of the rubber composites were conducted using 2 mm thick rubber sheets in an X-ray diffractometer (PANalytical XpertPro, Malvern, Worcestershire, UK) with CuKα (0.154 nm) radiation. The scans were performed by varying Bragg’s angles (2θ) from 10° to 30° at a scanning rate of approximately 4° per minute.

### 2.6. Scanning Electron Microscopic Studies

The morphologies of the tensile fractured surfaces were investigated using a scanning electron microscope (FE-SEM, S-4800, Hitachi, Tokyo, Japan). Prior to SEM analysis, the surfaces were coated with platinum using a sputtering machine.

### 2.7. Thermogravimetric Analyses

Thermogravimetric analyses of the rubber compounds were conducted using a TA instrument (SDT Q600, New Castle, DE, USA) with a temperature range of 20–800 °C in a nitrogen atmosphere.

### 2.8. Swelling and Thermodynamic Studies

To study the solvent swelling characteristics of the different rubber composites, known amounts of vulcanized rubber were immersed in toluene for 7 days to reach equilibrium at room temperature. Afterward, the samples were removed, and the weight of the swollen sample was measured. The weight of the swollen solvent was determined by subtracting the initial weight from the weight of the swollen sample. Finally, the swelling index was calculated based on the weight of the swollen solvent relative to 100 g of rubber compound.

The swelling of the solvent by rubber is a thermodynamic process where no internal energy change is assumed [50]. Hence, the Gibbs equation for thermodynamic equilibrium can be expressed as shown in Equation (1):

(1) ∆G=−T∆S

where ΔG represents the Gibbs free energy, and ΔS represents the conformational entropy change due to solvent swelling. Since mixing filler with rubber is also related to an entropic change, the change in entropy from unfilled to filled rubber may be attributed to filler–polymer interactions. ΔG can be measured according to the Flory–Huggins equation [51], as shown in Equation (2):

(2) $$\Delta G=RT\left[ln\left(1-V_{r}\right)+V_{r}+\chi V_{r}^{2}\right]$$

where R is the gas constant, T is the temperature in the absolute scale, V_r_ is the volume fraction of rubber in the swollen specimen, and χ is the interaction parameter in the rubber–solvent system. The V_r_ values were obtained from equilibrium swelling data using Equation (3):

(3) $$V_{r}=\left\{(w_{r}/d_{r})\right\}/\{(w_{r}/d_{r})+(w_{s}/d_{s})\}$$

where w_r_ and w_s_ are the weights of rubber and solvent, respectively, and d_r_ and d_s_ are the densities of rubber and solvent, respectively. The density of the solvent (toluene) was 0.87 g/cm^3^, and the χ values for the NR–toluene and NBR–toluene systems were taken as 0.378 and 0.425, respectively, from existing literature [52,53].

### 2.9. Strain Sensing and Electromechanical Actuation Studies

The piezoresistive strain sensing behavior of the rubber composites was studied using a Keithley 2634B source meter under uniaxial tensile strain, employing dumbbell-shaped specimens. Uniform strain was applied using a tensile testing machine, and the corresponding resistances were recorded. The relative resistance was calculated using the formula presented in Equation (4):

(4) $$Relativeresistance(\%)=(R-R_{0})\times 100/R_{0}$$

where R is the resistance at a certain strain, and R₀ is the initial resistance.

The capacitance of the composites was measured using cylindrical samples (d = 20 mm, h = 10 mm), with two electrodes placed on opposite sides.

**Figure 2:**
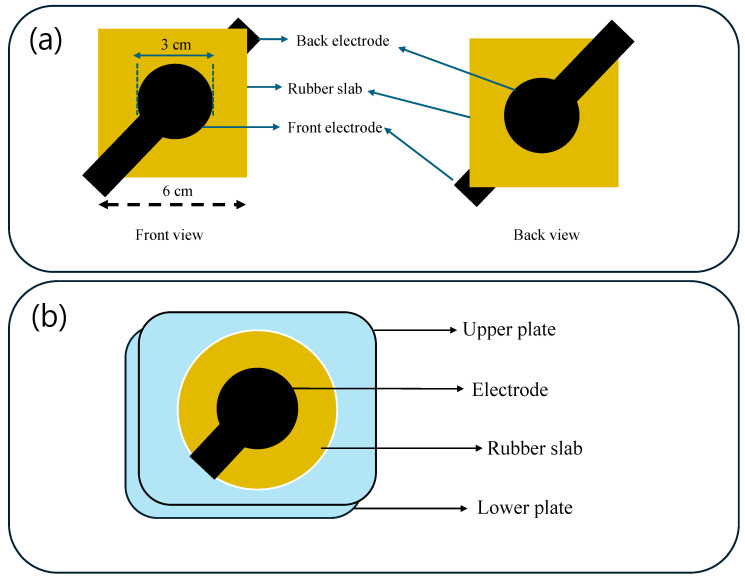
(**a**) Schematic of actuator samples and (**b**) schematic of actuator.

The dielectric actuation performances of different unfilled rubbers were studied using 0.1 mm thick electrodes, based on 2 phr CNT and room temperature vulcanized silicone rubber, which were painted on both sides of 1 mm thick, 6 cm × 6 cm square-shaped test specimens, as described elsewhere [54,55]. The actuation displacement under DC voltages was measured using a laser sensor (Opto NCDT 1302, Micro-Epsilon Messtechnik, Ortenburg, Germany). The schematics of the actuator sample and the actuator setup are provided in Figure 2a,b.

The equivalent electromechanical pressure (P_eq_), which is twice that of the electrostatic pressure, was determined according to the formula presented in Equation (5):

(5) $$P_{eq}=\varepsilon_{0}\varepsilon_{r}V^{2}/z^{2}$$

where ε_0_ is the vacuum permittivity (8.85 × 10⁻^12^ F/m), ε_r_ is the dielectric constant of the rubber, V is the applied voltage, and z is the thickness of the rubber slab during deformation. The value of z was calculated by considering the change in the diameter of the electrode, assuming no net volume change after actuation. The ε_r_ values of unfilled NR and NBR were taken as 2.70 and 8.28, respectively, at room temperature and 1 kHz frequency, as reported in the literature [56,57].

## 3. Results and Discussion

### 3.1. Mechanical and Physical Properties

**Figure 3:**
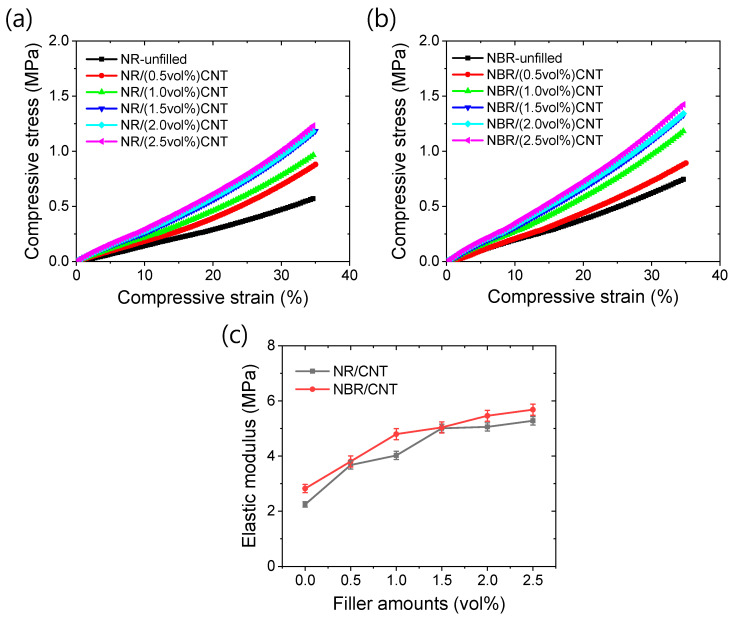
Compressive mechanical properties. (**a**) Stress–strain curves of NR-based nanocomposites, (**b**) stress–strain of NBR-based nanocomposites, and (**c**) elastic modulus.

The compressive mechanical properties of the rubber compounds are presented in Figure 3a–c. Figure 3a,b shows the compressive stress–strain relationships for NR- and NBR-based rubber composites. From these figures, it is evident that the stress value increases with increasing strain and CNT content. However, no significant increase in the ultimate stress values at 35% compressive strain is observed beyond 1.5 vol% CNT content. The substantial increase in ultimate stress values with respect to the filler content up to 1.5 vol% CNT may be attributed to the development of filler–rubber interfacial interactions, which become saturated at this level [58]. The slight increase in ultimate stress values beyond this critical filler content suggests that no significant additional filler–rubber networks are developed, and filler aggregation may occur beyond 1.5 vol% CNT content. It is believed that, above a certain threshold, the filler particles are weakly adhered to each other via van der Waals forces, resulting in minimal additional stress in the rubber composites. This hypothesis is further supported by the elastic modulus values presented in Figure 3c. From this figure, it is clear that the elastic modulus increases more rapidly up to 1.5 vol% CNT loading in the rubber composites. Beyond this threshold, there is only a slight increase in the elastic modulus due to the rigidity of the CNT, which is much higher compared to the rubber matrix.

**Figure 4:**
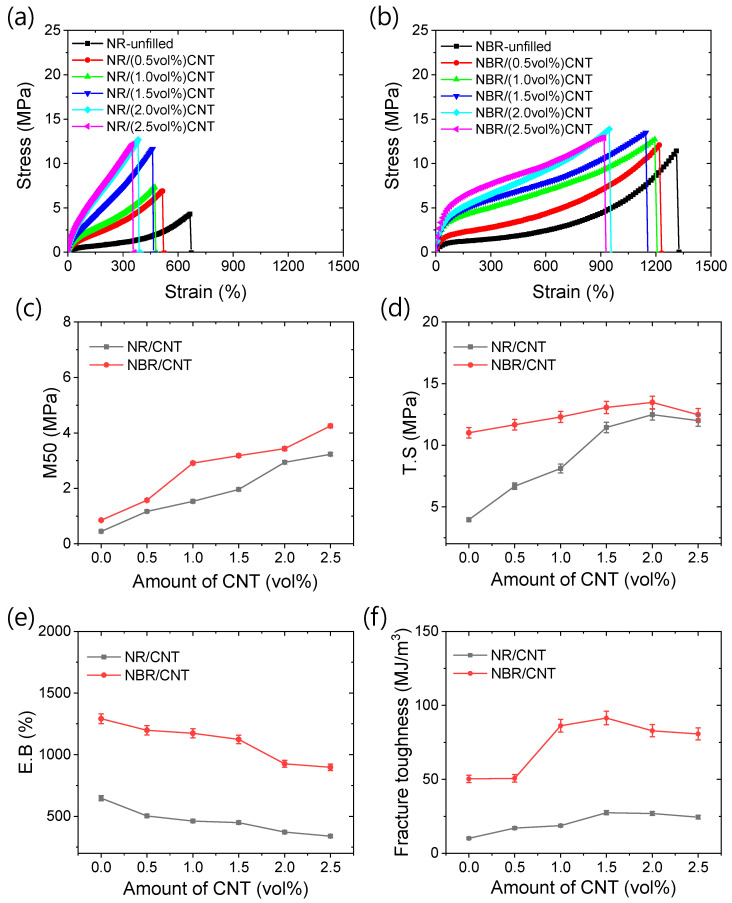
Tensile mechanical properties of rubber nanocomposites. (**a**) Stress–strain of NR-based composites, (**b**) stress–strain of NBR-based composites, (**c**) M50, (**d**) T.S, (**e**) E.B, and (**f**) fracture toughness.

The tensile mechanical properties of the rubber compounds are presented in Figure 4a–f. Figure 4a and Figure 4b show the tensile stress–strain curves for NR- and NBR-based rubber compounds, respectively. The stress values of the unfilled rubber are significantly enhanced by the addition of reinforcing CNT in the rubber matrix. The stress–strain curves also reveal that there are no significant enhancements in the stress–strain slopes after the addition of 2 vol% CNT. This suggests that rubber–filler interfacial interactions are optimized at this filler concentration. A slight enhancement in the stress–strain slope beyond the optimum filler amount may be attributed to filler–filler interactions, which could result from anisotropic filler distribution [58]. Interestingly, when comparing the stress–strain slope values of different rubber systems, the slope values are higher for NBR systems than for NR systems at lower strain amplitudes. However, the slope value for NBR systems decays more noticeably than that for NR systems, as strain increases. This reduction in the stress–strain slope at a higher strain may be due to the breakdown of filler networks [59].

From Figure 4c, it can be observed that the moduli at 50% strain (M50) are higher for NBR-based composites compared to NR-based composites at similar filler contents. However, at strains exceeding 300%, the moduli of NBR-based composites are lower than those of NR-based composites. These results suggest that the addition of CNTs to NBR rubber primarily enhances the viscous modulus rather than the elastic modulus, likely due to weak physical bonding, which degrades more easily with increased strain [59]. This hypothesis is further supported by the dynamic mechanical studies presented in a later section. Since physical bonding, such as that through van der Waals forces, diminishes more rapidly with increasing deformation, it can be inferred that the CNT filler is primarily bound by a higher number of physical bonds. As a result, the modulus is higher at lower deformation but degrades more quickly with increasing strain. In contrast, the NR matrix is likely to form a higher number of chemical bonds with the filler, as discussed later in the dynamic mechanical and thermal properties sections. These chemical bonds are more stable and do not degrade as easily as physical bonds, allowing the NR-based composites to retain their strength at higher strains. Additionally, the stress–strain slope may degrade up to a certain strain and then increase again due to the strain-induced crystallization of the rubber chains.

The specific tensile mechanical properties, including tensile strength (T.S.), elongation at break (E.B.), and fracture toughness, for the composite materials are shown in Figure 4d, Figure 4e, and Figure 4f, respectively. From Figure 4d, it is evident that NBR-based composites exhibit higher tensile strength than NR-based composites. Additionally, the NBR-unfilled compound demonstrates higher tensile strength than the NR-unfilled compound. However, the tensile strength values increase more rapidly with the addition of filler in the NR systems. In both rubber systems, the tensile strength reaches a maximum at 2 vol% CNT content, indicating that uniform filler dispersion is optimized at this level. The elongation properties are presented in Figure 4e, where it is observed that the addition of filler reduces the polymer chain stretchability. These results suggest that the filler introduces additional forces, such as chemical or physical bonding, which restrict the movement of the rubber chains. More importantly, from Figure 4f, it can be seen that fracture toughness increases rapidly with the addition of filler. The maximum toughness values are achieved at 1.5 vol% CNT content, and beyond this amount, the fracture toughness slightly decreases. It is clear that the tensile strengths of NR- and NBR-based composites at 2 vol% CNT content reach maximum values of 12.48 MPa and 13.47 MPa, which are approximately 216% and 22% higher, respectively, than those of the neat rubbers. Similarly, the fracture toughness values of NR- and NBR-based nanocomposites at 1.5 vol% CNT content reach peak values of 27.46 MJ/m^3^ and 91.43 MJ/m^3^, which are 169% and ~82% higher, respectively, than those of the neat rubbers. These results suggest that both types of rubber exhibit a similar trend in mechanical properties with respect to the filler amounts, with the optimal values for tensile mechanical properties obtained at 1.5 to 2 vol% CNT content. Beyond this point, the filler particles tend to aggregate significantly, leading to a reduction in mechanical properties.

**Figure 5:**
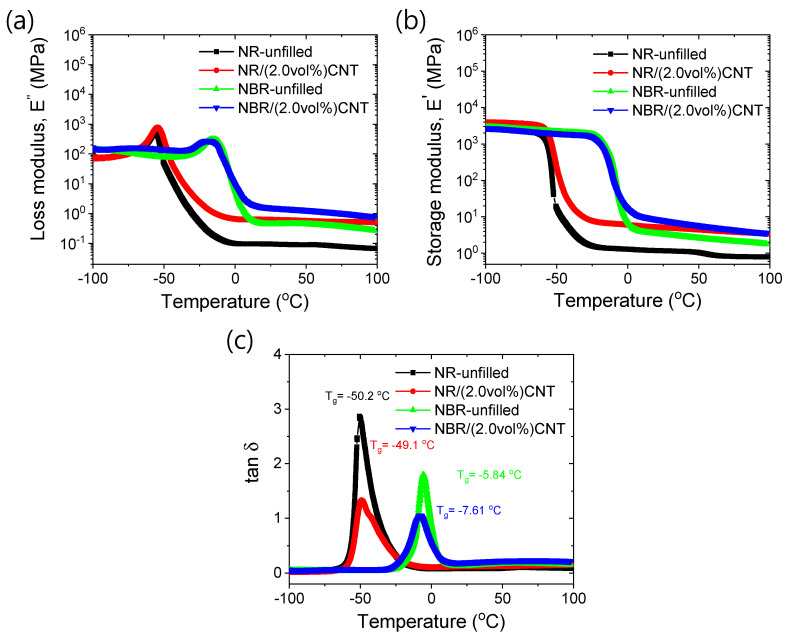
Dynamic mechanical properties. (**a**) Loss modulus, (**b**) storage modulus, and (**c**) tan δ.

To better understand the interfacial interactions between rubber and CNT, the dynamic mechanical analyses of unfilled rubber and 2 vol% CNT-containing rubber composites were conducted. The different dynamic mechanical properties are presented in Figure 5a–c. From Figure 5a, it can be seen that the loss modulus reaches its maximum value at the glass transition temperature (T_g_). As the temperature increases beyond T_g_, the loss modulus decreases. The loss modulus after T_g_ is higher in the 2 vol% CNT-filled rubber composites compared to the unfilled rubber systems. This may be due to the enhanced viscosity resulting from rubber–filler interfacial interactions. Higher loss modulus values in neat NBR compared to neat NR in the rubbery state suggest that stronger polymer–polymer interactions exist in NBR. To confirm a more homogeneous filler dispersion, the solvent blending technique was employed in this study, which involves solvent-diluted polymer chains. The addition of CNT filler significantly improves the loss modulus values, likely due to the development of filler–rubber interactions. Similarly, the storage modulus in Figure 5b increases with the addition of the rigid filler. The loss factor (tan δ) plots for different rubber compounds are shown in Figure 5c. It was found that the height of the tan δ peak at T_g_ is reduced with the addition of CNT. However, a slight increase in T_g_ suggests that additional chemical bonds may form between the active sites of CNT and the NR matrix, or that CNT may enhance the number of sulfur crosslinks, as NR has a higher number of crosslinking sites than NBR. In the case of NBR, a slight decrease in T_g_ indicates that CNT may interact with the rubber through van der Waals-type physical forces, which enhances the modulus values. For strong filler–polymer interactions, the height of the tan δ peak always decreases but does not always result in an increase in T_g_ compared to the neat polymer [60]. From the loss factors at higher temperatures, it can be inferred that NBR-based composites exhibit higher heat buildup properties than NR-based composites. Therefore, for dynamic applications, such as in tires, CNT-based NR composites may be more preferable.

**Figure 6:**
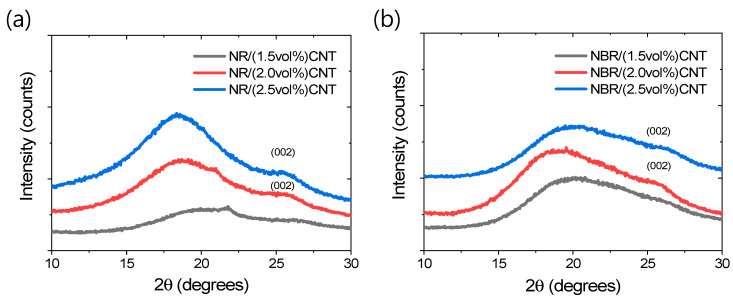
XRD patterns of rubber composites. (**a**) NR-based and (**b**) NBR-based.

To investigate the state of filler distribution in the rubber matrix, XRD and SEM studies were performed. The XRD patterns of different rubber composites are provided in Figure 6a,b. The broad XRD peak starting from 15° to 30° can be attributed to the amorphous rubber phase [61]. From Figure 6a,b, it is evident that, as the amount of CNT increases, a stronger 002 plane peak at 26.426° for CNT is observed. The XRD curves show that below 2 vol%, there is no intense 002 plane peak. However, with increasing filler amounts starting from 2 vol%, more intense 002 plane peaks are obtained for both rubber composites. The intense 002 plane peak may be attributed to the aggregation of CNT particles in the rubber matrix [61]. The smaller increments in mechanical properties and thermodynamic parameters (discussed in a later section) at filler contents higher than 1.5 vol% suggest filler aggregation in the rubber matrix. Furthermore, the higher intensity of CNT peaks in NR systems compared to NBR systems after the optimum filler amount may indicate greater filler particle aggregation. This could be due to the lower physical interactions of NR chains with CNTs, which promote the more pronounced aggregation of the filler particles.

**Figure 7:**
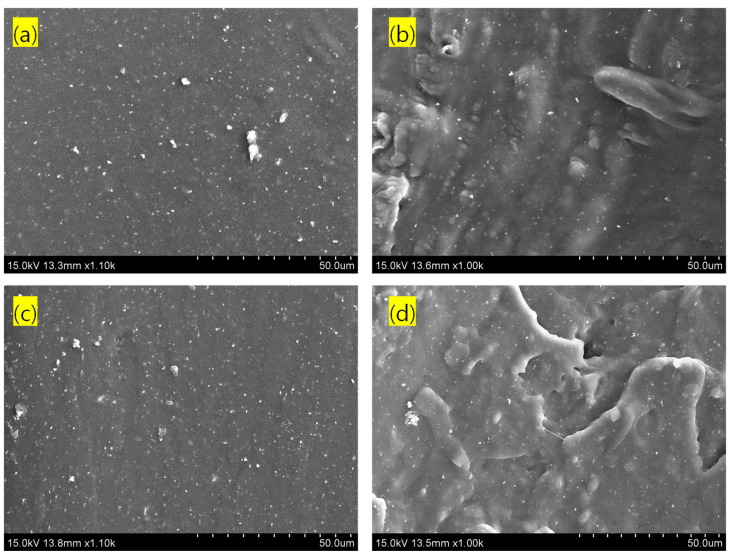
SEM images of tensile fractured surfaces. (**a**) NR-unfilled, (**b**) NR/(2.0 vol%)CNT, (**c**) NBR-unfilled, and (**d**) NBR/(2.0 vol%)CNT.

The SEM images of unfilled and filled rubber composites are provided in Figure 7a–d. Comparing Figure 7a with Figure 7b and Figure 7c with Figure 7d, it is evident that both rubbers exhibit excellent filler dispersion, with the rubber additives well distributed in the 2 vol% CNT-containing composites. The rough structures of the fractured surfaces in the SEM images (Figure 7b,d) clearly indicate that the composites were toughened after the addition of reinforcing CNT to the neat rubber (Figure 7a,c).

### 3.2. Thermal Degradation Behaviors

**Figure 8:**
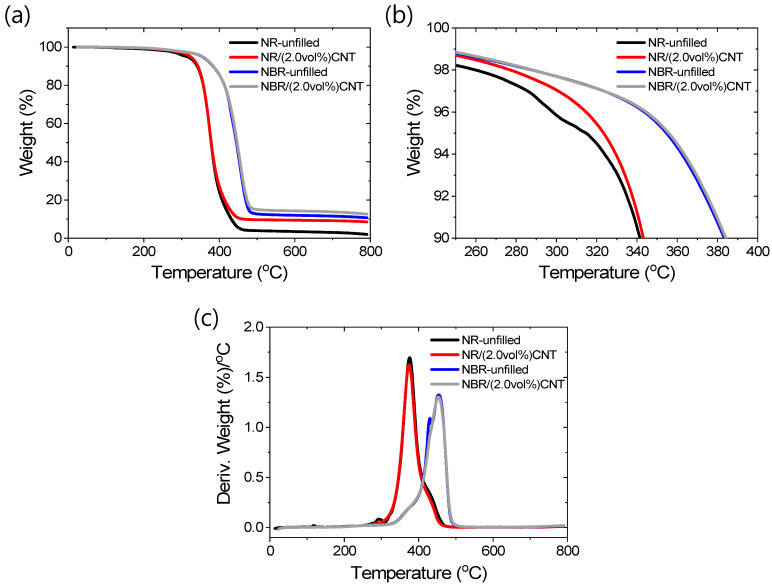
Thermal degradation behavior of rubber compounds. (**a**,**b**) TGA curves and (**c**) DTA curves.

The thermal properties of selected rubber compounds were studied, and the results are provided in Figure 8a–c. Detailed discussions of different fragments of thermally degraded NR and NBR can be found elsewhere [62,63,64,65]. From the TGA curves in Figure 8a,b, it is evident that CNT at 2 vol% significantly enhances the initial degradation temperature (~3% weight loss) of NR but has a negligible effect on NBR. However, the initial degradation temperature of NBR-based compounds is much higher compared to NR-based compounds. Additionally, the char residues of NBR-based compounds are comparatively higher than those of NR-based compounds. These results indicate that NBR inherently has higher thermal stability but exhibits less chemical interaction with CNT. On the other hand, NR has lower thermal stability, but its interaction with CNT, likely through stronger chemical bonding, improves the thermal stability by increasing the initial degradation temperature and char residue. It is evident that 2 vol% CNT can enhance the initial degradation temperature by approximately 15 °C in the NR/(2.0 vol%) CNT composite. The derivative thermogravimetry (DTG) curves in Figure 8c suggest that NBR-based compounds have higher thermal stability and a lower degradation rate compared to the NR-based compound [62,63,64,65].

The DMA analyses reveal that NR exhibits stronger chemical interactions with CNTs, forming chemical bonds between the rubber and filler, which results in a shift of T_g_ to a higher temperature. In contrast, the T_g_ of CNT-containing NBR composites shifts to a lower temperature, while the height of the tan δ peak decreases, suggesting that physical-type interactions exist between NBR and the filler. This finding is further supported by the TGA analyses, which confirm that NR chains are grafted with stronger chemical bonds to CNT particles, enhancing the thermal stability of the composite compared to the physically bonded CNT in NBR.

Regarding the chemical structures, NR consists of polyisoprene units, while NBR contains butadiene and acrylonitrile units. The methyl group in NR is highly reactive [66], which may form chemical bonds with the sp3 or reactive carbon atoms on the CNT surface. Since the vulcanization reaction is primarily a radial-type reaction, curing additives generate radicals [66]. These radicals can interact chemically with the active sites of the rubber chains and CNT particles, forming chemical bridges between them. On the other hand, NBR contains nitrile groups that are chemically less reactive for crosslinking, and the backbone methylene protons cannot form crosslinks with the CNT particles, likely due to steric hindrance. However, due to its permanent dipole, NBR has more physical interactions, such as dipole–dipole and n–π interactions, compared to NR-based composites.

### 3.3. Swelling and Thermodynamic Properties

**Figure 9:**
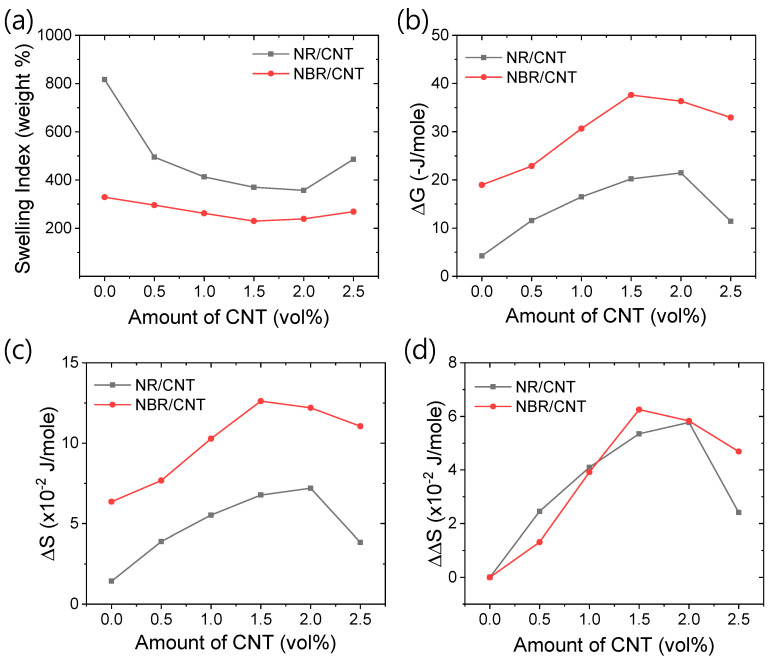
Characteristics of swelling and thermodynamic properties. (**a**) Swelling index, (**b**) ΔG, (**c**) ΔS, and (**d**) ΔΔS.

The swelling study is an essential and straightforward method for understanding the polarity of rubber and its intermolecular interactions [67]. The different swelling and thermodynamic properties are presented in Figure 9a–d. From Figure 9a, it is evident that neat NBR has a lower swelling index value than neat NR. This can be attributed to the polar nitrile group in NBR, which leads to higher dipole–dipole interactions, as well as other van der Waals interactions. Consequently, NBR exhibits stronger polymer–polymer interactions, resulting in lower swelling in toluene compared to NR. With the addition of CNT, NR shows a more pronounced reduction in its swelling properties compared to NBR. Composites with 1.5 to 2 vol% CNT content exhibit the lowest swelling index values. However, beyond 1.5 vol% CNT, the swelling index does not significantly decrease and may even increase with further CNT addition. Therefore, to achieve a reduced swelling index, the CNT content should not exceed 2 vol% in the rubber.

The types of interactions present in the rubber compounds are further clarified in Figure 9b–d. From Figure 9b,c, it is clear that the ΔG values are more negative, and the ΔS values are significantly higher for NBR-based composites compared to NR-based composites. From a thermodynamic perspective, this indicates that NBR-based composites have stronger total interactions, including polymer–polymer and filler–polymer interactions, compared to NR-based composites. The higher binding strength in NBR-based composites is also reflected in their higher fracture toughness values and thermal stabilities compared to NR-based composites. The contribution of filler to the total strength is determined by subtracting the ΔS value of unfilled rubber from that of filled rubber. Figure 9d represents the specific contribution of filler to the ΔS value by ΔΔS. It is evident from this figure that ΔΔS values are lower for NBR-based rubber composites compared to NR-based composites at filler contents below 1 vol%. However, the trend reverses at higher filler content. This could be due to higher polymer–polymer interactions controlling filler dispersion at lower filler concentrations, while at higher filler contents, the increased filler–polymer interactions contribute more to reinforcement. At higher levels of filler dispersion, the n–π stacking interactions between the nitrogen atom from the nitrile group in NBR and the π-electrons of CNT play a significant role in contributing to filler reinforcement [68]. From this figure, it is evident that the contribution of filler–polymer interactions sharply decreases with more than 2 vol% filler content. Therefore, the CNT filler content in the rubber should not exceed 2 vol%.

### 3.4. Electrical Responses of Rubber Nanocomposites

Rubber composites become electroactive upon the introduction of electrical conductivity. To achieve electrical conductivity in the rubber composite, the conducting filler content must reach the percolation threshold. In this study, significant electrical conductivity was observed in both NR- and NBR-based composites after the addition of 1 vol% CNT. Based on the mechanical properties of the rubber composites, it can be concluded that the filler content should be kept below 2 vol% to obtain optimal mechanical performance. Therefore, composites containing 1 to 2 vol% CNT were further investigated for their strain-sensing properties.

**Figure 10:**
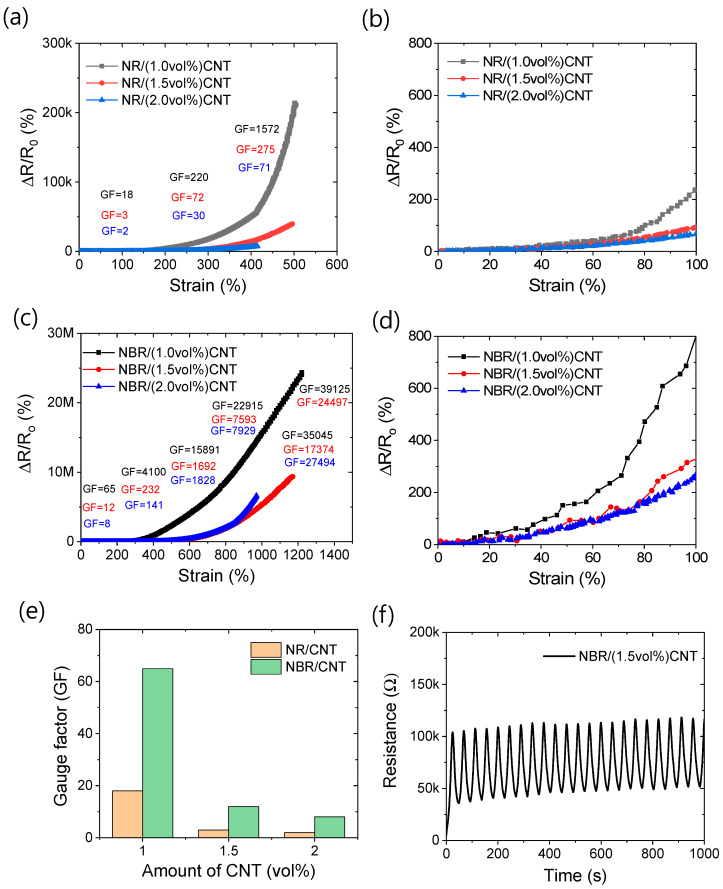
Strain sensing properties. (**a**) Strain sensitivity curves of NR-based composites with different gauge factors with interval of 200% elongation, (**b**) strain sensitivity curves of NR-based composites with 100% elongation, (**c**) strain sensitivity curves of NBR-based composites with different gauge factors with interval of 200% elongation, (**d**) strain sensitivity curves of NBR-based composites with 100% elongation, (**e**) gauge factors within 0–200% strain, and (**f**) real-time strain sensing at 200% cyclic strain.

The strain-sensing behaviors of the rubber composites are shown in Figure 10a–d, with gauge factor (GF) values calculated for the strain range (Δε) of 200%, excluding the end ranges. From these figures, it is clear that the relative resistance change increases with increasing strain amplitude. These figures also demonstrate that the linearity in the relative resistance changes with strain decreases as the strain increases. However, below 100% strain, the strain sensitivity curves maintain better linearity (Figure 10b,d). Additionally, it is evident that the linearity of strain sensitivity can be enhanced at the expense of reduced sensitivity with higher filler amounts. Figure 10e shows that, for similar strain ranges (0% ≤ Δε ≤ 200%), NBR-based composites exhibit higher GFs compared to NR-based rubber composites. From this figure, it is clear that the GF values of NBR-based composites are significantly higher than those of NR-based composites at similar filler amounts. The NBR/(1 vol%) CNT composite shows a GF of 65 at the 0% ≤ Δε ≤ 200% strain range, which is 260% higher than the GF of NR/(1 vol%) CNT. Higher GFs are obtained at lower filler amounts, because the highest resistance change occurs within the filler percolation region. As a result, higher GFs can be achieved at this level. Above the filler percolation threshold, sensitivity or GF may decrease, but linearity can be enhanced [33]. The gauge factor can reach as high as 39,125 at maximum deformation for the 1 vol% CNT-containing NBR composite. However, larger deformations or higher working strains should be avoided due to the potential for high hysteresis loss [32,33].

Figure 10f represents typical strain sensitivity at 200% cyclic strain for NBR composites containing 1.5 vol% CNT. Although the sensitivity curves are not perfectly linear, the figure demonstrates that the sensitivity peaks exhibit good repeatability, which can be useful for measuring strain sensitivity more accurately [37,69,70]. Compared to NR-based composites, NBR-based composites show considerably better strain sensitivity.

It is well-established that the strain sensitivity of polymer composites depends on several factors, with the characteristics of the polymer materials, fillers, and fabrication techniques being the most critical. For reliable strain sensors, the strain sensitivity and detection limit are two of the most important factors. For clarity, a comparison table (Table 2) is provided, showing that strain-sensing composites were made by in situ filler dispersion in the polymer matrix. From this table, it is evident that both NR- and NBR-based CNT composites offer better gauge factors and superior working strain ranges compared to previously established strain-sensing composites [32,33,71,72].

To investigate the origin of the observed behaviors, we measured the capacitance of the rubber composites, as presented in Table 3. From this table, it is evident that the capacitance of the composites significantly increases with the addition of an optimal amount of CNT. However, beyond this optimal CNT concentration, the capacitance may decrease or even disappear due to reduced resistance and higher electrical conductivity, which prevents charge accumulation. The table also shows that, at similar filler concentrations (typically 0.5 vol%), the capacitance of the NBR-based composite is more than 10,000 times higher than that of the NR-based composite. This substantial difference in capacitance can be attributed to the significantly different dielectric constant values of the two rubbers.

It can be inferred that, as the strain increases, the conductivity of the composite decreases, indicating strain sensitivity. Several mechanisms contribute to this strain sensitivity [37]. Among them, the disconnection of filler particles is the primary reason for the change in conductivity with increasing strain. It is well known that, instead of direct contact between filler particles, electrons can be transported via the tunneling effect over a critical distance between two filler particles [73]. This critical distance is covered by the rubber matrix. Due to the higher dielectric constant of NBR, electron transport is less permeable compared to NR. As a result, even slight deformation can significantly alter the conductivity of the composites, leading to a higher resistance change in NBR-based composites than in NR-based composites at similar filler contents.

Since tunneling resistance is more sensitive to strain-induced contact resistance, conductivity changes more rapidly at higher strains, making the composites more sensitive and leading to an increase in the gauge factor (GF). At similar strain levels, the tunneling resistance of NBR-based composites is believed to change more significantly due to the higher dielectric constant of NBR compared to NR, resulting in higher GF values for NBR-based composites at similar filler amounts. The higher strain sensitivities at lower filler amounts are attributed to the greater change in tunneling resistance. With a lower filler content, maximum conductivity arises from the tunneling effect. However, a lower filler content shows less linearity in the sensing curves, suggesting a nonlinear change in conductivity with strain. Therefore, to obtain a reliable strain-sensitive composite, both the rubber type and filler amount should be carefully considered.

**Figure 11:**
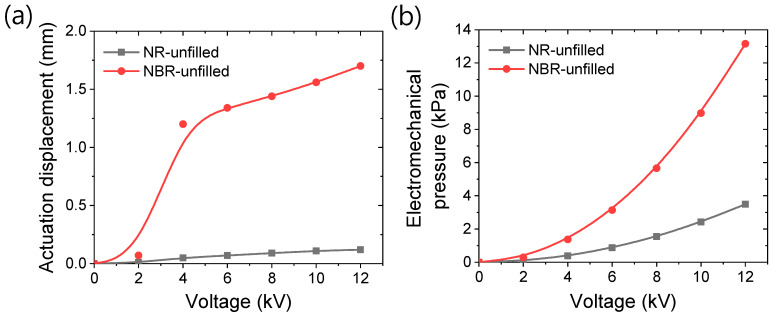
Variation in electromechanical properties in dielectric rubbers. (**a**) In-plane actuation displacement with respect to applied voltage, and (**b**) electromechanical pressure with respect to applied voltage.

The actuation performance of dielectric rubber depends on several factors, including mechanical properties, but it is primarily influenced by the applied voltage and the dielectric constant of the rubber [74,75,76]. Among these, the dielectric constant is the most important factor for generating higher mechanical properties. The actuator performances of different unfilled rubber systems are shown in Figure 11a,b.

From Figure 11a, it is evident that the in-plane electrode displacement values are higher in unfilled NBR compared to NR. This figure also shows that, after reaching the percolation voltage, a significant displacement can be observed at higher voltages. This result indicates that NBR rubber is more sensitive to the electric field compared to NR. In the presence of an electric field, an induced dipole moment is generated. These dipoles tend to orient in the direction of the applied electric field, causing actuation displacement. From this perspective, NBR, being a polar rubber, has better orientation and attraction between the dipoles, resulting in larger actuation compared to the nonpolar NR. Suresh et al. found that the chemical microstructure plays a vital role in improving the dielectric constant, and NBR has a higher dielectric constant than other rubbers [75].

From Figure 11a, it can be seen that a threshold voltage is needed to achieve higher actuation displacement. This might indicate that the nitrile groups in NBR are anisotropically distributed, with stronger dipole–dipole interactions, enhancing the inherent mechanical properties, as observed in the previous sections. Thus, a threshold voltage is required to overcome these interactions and align the dipoles in the direction of the applied electric field. The calculated electromechanical pressure generated in the actuators is provided in Figure 11b. From this figure, it is clear that the NBR-based actuator produces higher electromechanical pressure at similar applied voltages. For example, at a 12 kV applied voltage, the NBR-based actuator produces approximately 1317% higher displacement and 276% higher pressure compared to the NR-based actuator. The higher electromechanical pressure in the NBR-based actuator indicates that, for dielectric actuators, the rubber should be more polar with a higher dielectric constant.

Similar to unfilled dielectric elastomers, CNT-based elastomer composites may also have electromechanical actuation properties, but they were not studied in this work. It is believed that, due to their higher electrical conductivity, CNT-based composites can easily reach the breakdown voltage, thereby losing their actuation performance.

**Figure 12:**
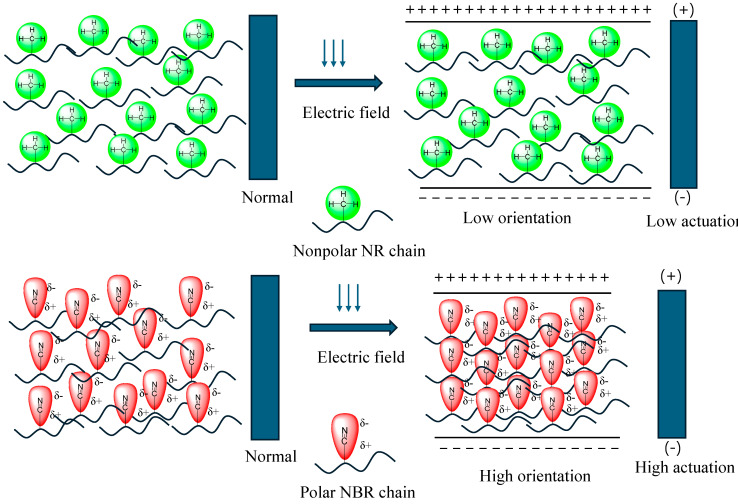
Possible mechanism of electromechanical actuation in different functional rubbers.

Based on the above results, a mechanism of behavior for different rubbers in an electric field can be presented, as shown in Figure 12. NBR contains numerous nitrile groups that introduce polarity to the rubber, and these polar groups are in an isotropic state in the absence of an electric field. In the presence of an electric field, these groups tend to orient. Since these groups exist as side chains, they are flexible and can easily orient in the direction of the applied electric field. At a threshold voltage, the flexible nitrile groups almost completely orient, and thereafter, they come closer, causing actuation in the rubber slab. On the other hand, NR is a nonpolar rubber with a low dielectric constant compared to NBR. Hence, the induced dipoles are very weak in the presence of applied electric fields, resulting in lower actuation properties.

## 4. Conclusions

The aim of this article was to investigate the role of functionality in diene rubbers concerning mechanical reinforcement and electromechanical activities in carbon nanotube-based electroactive rubber composites. Static and dynamic mechanical studies suggest that CNT strongly reinforces the rubbers at an optimum amount of approximately 2 vol% in both NR- and NBR-based composites. From the elastic modulus, tensile strength, and fracture toughness values, it is evident that CNT has higher reinforcing efficiency toward NR, with more significant changes in these properties compared to NBR. At 2 vol% CNT content, the glass transition temperature value of the NR-based composite shifted to a higher temperature, and the thermal stability of the composite improved, with about a 15 °C enhancement in the initial degradation temperature compared to the NBR-based composite. X-ray diffraction and SEM analyses of the rubber composites indicate that CNT particles form aggregates above 1.5 vol% CNT content, with this tendency being more pronounced in NR-based composites.

Thermodynamic studies also reveal that NR-based composites show greater changes in thermodynamic parameters at lower concentrations of CNT, but the changes decrease with increasing CNT content compared to NBR-based composites. From the higher changes in mechanical properties, increased glass transition temperature, improved thermal stability, and enhanced thermodynamic parameters with respect to unfilled rubber systems, it can be concluded that CNT forms stronger chemical bonds with NR rather than physical bonds with NBR.

The significant improvements in mechanical and dynamic mechanical properties with small amounts of CNT suggest that the composites can be useful for mechanical applications. Since CNT filler particles are attached to NR chains via stronger chemical bonds, NR-based composites may be more suitable for dynamic mechanical applications with lower heat build-up properties. Although a certain amount of chemical bonding between rubber and filler may form in NR-based composites, the better elasticity in NBR-based composites could be due to its higher overall bonding capacity, including both physical and chemical interactions.

Strain-sensing activities of the rubber composites suggest that NBR-based composites exhibit higher strain-sensing properties, with higher gauge factor or sensitivity values and a wider detection limit compared to NR-based electroactive composites at similar CNT contents. The best strain-sensitive composite, with the highest gauge factor values, can be obtained with 1 vol% CNT in the NBR-based composite. However, to achieve better linearity in the strain sensitivity with strain amplitude, 1.5 vol% CNT is necessary in the NBR-based strain-sensing composite. The strain sensor made from 1.5 vol% CNT and NBR rubber maintains its efficiency without significant loss up to 200% cyclic stretching and releasing.

The higher strain sensitivities in NBR-based composites compared to NR-based composites are likely due to the stronger electronic tunneling effect in the former. Due to the presence of polar functional groups and a higher dielectric constant, NBR demonstrates high strain sensitivity, significant actuation, and strong electromechanical pressure—260%, 1317%, and 276% higher, respectively, compared to NR. Hence, for fabricating high-performance electroactive rubber composites, selecting polar NBR with a high dielectric constant value may be more advantageous than using nonpolar NR. Moreover, as electrical energy is converted to mechanical energy as a result of actuation, these rubber composites, either with this filler or hybridized with other fillers, could be useful for converting mechanical energy into electrical power. Therefore, high-performance NBR-based composites have potential applications for producing clean energy from renewable resources in the future.

## Figures and Tables

**Table 1:** Mix compositions of different rubber composites.

Formulation	Masterbatch (g)	Amount of CNT in vol%
NR-unfilled	100	-
NR/(0.5 vol%)CNT	100	0.5 *
NR/(1.0 vol%)CNT	100	1
NR/(1.5 vol%)CNT	100	1.5
NR/(2.0 vol%)CNT	100	2
NR/(2.5 vol%)CNT	100	2.5
NBR-unfilled	100	-
NBR/(0.5 vol%)CNT	100	0.5
NBR/(1.0 vol%)CNT	100	1
NBR/(1.5 vol%)CNT	100	1.5
NBR/(2.0 vol%)CNT	100	2
NBR/(2.5 vol%)CNT	100	2.5

* To calculate the amounts of CNT in volume %, the densities of the unfilled rubbers were considered: 0.92 g/cm^3^ for NR and 0.95 g/cm^3^ for NBR.

**Table 2:** Sensitivity, working strain, and prepared methods comparison of CNT-based composite type strain sensors.

Composites	Fillers	Gauge Factor	Working Strain	Preparation Method	References
Epoxy resin/CNT	CNT	6	0.6–14%	Solvent casting with sonication and drying	[71]
PMVS/CNT/CB	CNT/CB	10	0–60%	Dry mixing in two-roll mill and vulcanized at 170 °C	[72]
RTV-SR/CNT/MoS_2_	CNT/MoS_2_	25.95	100–155%	Solution blending and vulcanization at ~25 °C (room temperature)	[32]
SBR/CNT	CNT	58.79	100–150%	Solvent blending and vulcanization at 150 °C	[33]
NR/CNT	CNT	1572	400–505	Solvent blending and vulcanization at 150 °C	Present work
NBR/CNT	CNT	39,125	1000–1220%	Solvent blending and vulcanization at 15 0°C	Present work

(PMVS: polymethylvinylsiloxane; RTV-SR: room temperature vulcanized silicone rubber; SBR: styrene butadiene rubber).

**Table 3:** Volume capacitance of different rubber composites.

Formulation	Capacitance (F/cm^3^)
NR-unfilled	0.0095 × 10^−9^
NR/(0.5 vol%)CNT	0.1338 × 10^−9^
NR/(1.0 vol%)CNT	15.9235 × 10^−9^
NR/(1.5 vol%)CNT	9.6306 × 10^−6^
NR/(2.0 vol%)CNT	6.5159 × 10^−6^
NR/(2.5 vol%)CNT	-
NBR-unfilled	0.0095 × 10^−9^
NBR/(0.5 vol%)CNT	1.3949 × 10^−6^
NBR/(1.0 vol%)CNT	-
NBR/(1.5 vol%)CNT	-
NBR/(2.0 vol%)CNT	-
NBR/(2.5 vol%)CNT	-

## Data Availability

Data will be available based on request to the corresponding author.
